# Microparticle-Mediated Transfer of the Viral Receptors CAR and CD46, and the CFTR Channel in a CHO Cell Model Confers New Functions to Target Cells

**DOI:** 10.1371/journal.pone.0052326

**Published:** 2012-12-20

**Authors:** Gaëlle Gonzalez, Cyrielle Vituret, Attilio Di Pietro, Marc Chanson, Pierre Boulanger, Saw-See Hong

**Affiliations:** 1 Université Lyon 1, UCBL-INRA-EPHE UMR-754, Retrovirus & Comparative Pathology, Lyon, France; 2 Equipe labellisée Ligue 2009, Unité BM2SI, UMR 5086 CNRS-Université Lyon 1, Institut de Biologie et Chimie des Protéines (FR 3302), Lyon, France; 3 Laboratory of Clinical Investigation III, Faculty of Medicine, Department of Pediatrics, Geneva University Hospitals and University of Geneva, Foundation for Medical Research, Geneva, Switzerland; 4 Institut National de la Santé et de la Recherche Médicale, Paris, France; Baylor College of Medicine, United States of America

## Abstract

Cell microparticles (MPs) released in the extracellular milieu can embark plasma membrane and intracellular components which are specific of their cellular origin, and transfer them to target cells. The MP-mediated, cell-to-cell transfer of three human membrane glycoproteins of different degrees of complexity was investigated in the present study, using a CHO cell model system. We first tested the delivery of CAR and CD46, two monospanins which act as adenovirus receptors, to target CHO cells. CHO cells lack CAR and CD46, high affinity receptors for human adenovirus serotype 5 (HAdV5), and serotype 35 (HAdV35), respectively. We found that MPs derived from CHO cells (MP-donor cells) constitutively expressing CAR (MP-CAR) or CD46 (MP-CD46) were able to transfer CAR and CD46 to target CHO cells, and conferred selective permissiveness to HAdV5 and HAdV35. In addition, target CHO cells incubated with MP-CD46 acquired the CD46-associated function in complement regulation. We also explored the MP-mediated delivery of a dodecaspanin membrane glycoprotein, the CFTR to target CHO cells. CFTR functions as a chloride channel in human cells and is implicated in the genetic disease cystic fibrosis. Target CHO cells incubated with MPs produced by CHO cells constitutively expressing GFP-tagged CFTR (MP-GFP-CFTR) were found to gain a new cellular function, the chloride channel activity associated to CFTR. Time-course analysis of the appearance of GFP-CFTR in target cells suggested that MPs could achieve the delivery of CFTR to target cells via two mechanisms: the transfer of mature, membrane-inserted CFTR glycoprotein, and the transfer of CFTR-encoding mRNA. These results confirmed that cell-derived MPs represent a new class of promising therapeutic vehicles for the delivery of bioactive macromolecules, proteins or mRNAs, the latter exerting the desired therapeutic effect in target cells via *de novo* synthesis of their encoded proteins.

## Introduction

The extracellular milieu contains a vast family of cell-derived, particulate elements which are heterogenous in size, depending upon the shedding process, cell type and cellular compartments from which they are issued. According to the most recent and generally accepted definition, extracellular microparticles (MPs) are membrane-derived vesicles with diameter ranging from 100 to 500 nm, which are released from virtually all cell types (reviewed in [1]–[5]). Extracellular release of MPs occurs in response to certain stress conditions [6], [7] or pathological processes [8]–[10]. However, MPs have also been shown to be released spontaneously and physiologically, and are now considered as key elements in the cell-to-cell communications [3], [11]–[13]. This include angiogenesis [14], blood coagulation [15], [16], infection by HIV-1 [17] and other viruses [4], carcinogenesis [18], inflammation [19], vaccinology [20], and more generally in immunity [8], [21]–[23]. One particular case of MP contribution to immunological processes has been termed ‘trogocytosis‘ [24], [25].

In general, MPs carry with them membrane and cytosolic components specific of their cellular origin [26], including proteins and nucleic acids, such as mRNAs and microRNAs [7], [11], [27]–[30], and are capable of transferring their cargo to recipient cells [16], [29], [31]–[34]. During their extracellular release, MPs can also embark components which are foreign to the cells, such as nucleic acids, proteins or glycoproteins expressed transiently or constitutively by a plasmid or viral vector. The latter scenario is reminiscent of the process of virus or virus-like particles (VLPs) pseudotyping by foreign glycoproteins [35]–[40]. MPs are not only considered as circulating biomarkers for the molecular profiling of certain cancers [41], but their therapeutic potential as conveyors of bioactive factors, proteins, RNAs including miRNAs, is being evaluated for personalized medicine and for the treatment of a number of diseases and cellular dysfunctions [5], [7], [9], [11], [27], [30], [42].

In the present study, we developed a cellular model using Chinese hamster ovarian cells (CHO) to analyse the MP-mediated transfer of three human transmembrane glycoproteins with different degrees of structural complexity and cellular topology, CAR, CD46 and CFTR. CAR (coxsackie-adenovirus receptor) and CD46 (complement regulatory protein and pathogen receptor) are well-characterized type I membrane receptors of the Ig-like family of molecules, which carry a single transmembrane domain (monospanins). Both CAR and CD46 can act as cell receptors for different viruses. CAR has been identified as the cell receptor for the human adenovirus serotype 5 (HAdV5), and other members of species A, C, D, E and F [43]–[46]. CD46 acts as a cellular receptor for several viral pathogens, including measles virus [47] and members of human adenovirus species B1, B2 (among them HAdV35) and D [43], [48], [49], and also plays the role of cofactor in the inactivation of complement components C3b and C4b by serum factor I [50]. CD46 and CAR differ in their cellular localization: CD46 is localized at the apical membrane of epithelial cells [48], [50], [51], whereas CAR is localized at the tight junctions [52]. The CFTR (cystic fibrosis transmembrane conductance regulator) is a complex type III membrane glycoprotein, with large intracytoplasmic N- and C-terminal domains and twelve membrane-spanning domains constituting a transmembrane, cAMP-dependent chloride channel [53]–[57]. It is mainly present at the cellular apical surface of epithelial cells [58], [59], and localised in microdomains of the plasma membrane referred to as lipid rafts [60].

CHO cells have been shown to efficiently release MPs, even in the absence of stressing factors [17]. CHO cells were used to establish stable cell lines which constitutively express CAR, CD46 and CFTR (MP-donor cells). MPs derived from these cells were first tested for their capacity to incorporate CAR, CD46 or CFTR (MP packaging or pseudotyping), and secondly, for their competency in transferring the CAR, CD46, and CFTR proteins and their specific functions to naive CHO cells used as target cells (MP-recipient cells). We found that new biological functions associated to CAR, CD46 or CFTR were acquired by the target CHO cells after incubation with MP-CAR, MP-CD46 or MP-CFTR, respectively. Our results demonstrated that MPs have the therapeutic potential for the cell-to-cell transfer of bioactive molecules. The function(s) carried over by MPs could be direct, via MP-packaged therapeutic proteins, or indirect, via specific mRNAs, such as the mRNA encoding the wild-type CFTR for the correction of the defective chloride channel function in cystic fibrosis.

## Results

### Isolation and Recovery of MPs from CAR- and CD46-expressing CHO Cells

**Figure 1:**
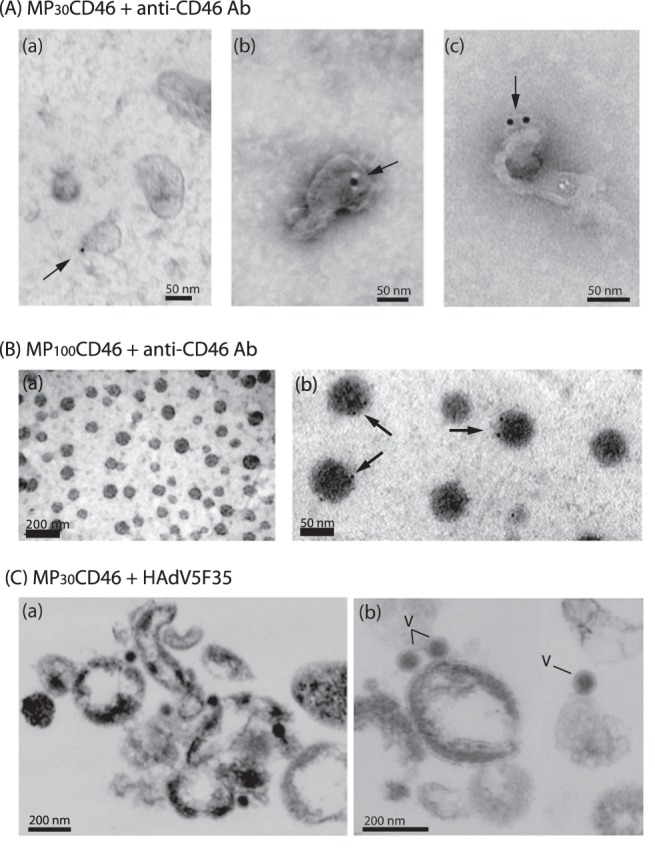
Electron microscopy (EM) of MPs **isolated from CHO-CD46 cells.** (**A**), Negative staining and immuno-EM of MP_30_CD46. (**B**), Negative staining (**a**), and immuno-EM (**b**) of MP_100_CD46. In (A) and (B, b), immunogold labeling was performed using anti-C46 antibody and 10 nm-gold tagged complementary antibody. MP-associated gold grains are indicated by arrows. (**C**), Ultrathin sections of pelletable complexes of MP_30_CD46-HAdV5F35, shown at low (a) and high (b) magnifications. Particles of HAdV5F35 vector (70–80 nm in diameter) in complex with MP_30_CD46 are indicated by the letter V.

The CHO-CAR and CHO-CD46 cells were derived from the parental CHO-K1 cell line to constitutively express and display CAR [44] and CD46 [61]–[63] at their surface, respectively. The recovery of MPs from stressed cells is usually higher than from nonstressed cells. However, since MPs issued from stressed cells could transfer stress signals and induce the apoptosis of recipient cells, our starting material was the culture medium of nonstressed CHO-CAR and CHO-CD46 cells. Extracellular MPs were recovered by a two-step ultracentrifugation procedure which separated MPs according to their size, consisting of a first sedimentation at 30,000×*g*, followed by a second at 100,000×*g*. Two populations were thus obtained, abbreviated MP_30_ and MP_100_, respectively (**Fig. S1**). The MP_30_ fraction contained large MPs characterized by their heterogeneity in shape and size, ranging from 50–500 nm in diameter, consistent with plasma membrane-shedded MPs (**Fig. 1A**). On the other hand, the MP_100_ population consisted of homogenous and regular particles, rather spherical and relatively small in size (50–100 nm; **Fig. 1B**), reminiscent of exosomes or exosome-like particles [2], [28]. Antibodies directed against human and mouse TSG101 and CD63, two surface markers of exosomes, were used in attempts to further characterize this population. However, no clear reaction was obtained with these antibodies on CHO cell lysates or MP pellets by Western blot or flow cytometry, respectively. Extracellular MPs released by CHO-CAR and CHO-CD46 cells were therefore differentiated only by their sedimentation properties, and referred to as MP_30_ and MP_100_ in the present study.

The yields of MPs spontaneously recovered from 10^7^ cells (CHO-CAR or CHO-CD46) ranged from 1×10^7^ to 3×10^7^ for MP_30_ after 72 h culture, and a similar recovery was obtained for MP_100_. After resuspension of the MP pellets in PBS, the total MP concentration (titer in physical MPs) of our working stocks usually ranged between 4×10^7^ to 7×10^7^ MPs/ml for MP_30_ or MP_100_, corresponding to total protein concentrations in the range of 700–850 ng/ml. Considering their size and composition heterogeneity, an average of 100 ng protein corresponded to a total number of 5×10^6^ to 7×10^6^ MP_30_ or MP_100_.

### Efficiency of Incorporation of CAR and CD46 by MPs

MPs isolated from CHO-CAR and CHO-CD46 cells by our two-step ultracentrifugation procedure were referred to as MP_30_CAR and MP_100_CAR, and MP_30_CD46 and MP_100_CD46, respectively. The proportion of CAR-positive and CD46-positive MPs was determined by flow cytometry using specific antibodies, as this technique detected the CAR and CD46 molecules exposed at the MP surface, and potentially active as adenoviral receptors. The mean value of the percentage of MP_30_CAR to total MPs was 5.8±1.4% (mean ± SEM, *n* = 3), corresponding to a mean bioactive MP titer of 2×10^5^ MP_30_CAR/ml, and only 2.8±1.2% for MP_100_CAR. On the other hand, the mean percentage of MP_30_CD46 to total MPs was 7.5±1.5% (viz. a mean bioactive MP titer of 5×10^5^ MP_30_CD46/ml), versus 2.2±1.3% for MP_100_CD46. Due to the higher proportion of MPs positive for CAR and CD46 glycoproteins in the MP_30_ populations, MP_30_CAR and MP_30_CD46 were used for the MP-mediated protein transfer experiments. By taking into account the value of the bioactivity titer of our MP_30_ stocks, the MP dose per target CHO cell ranged from 5 to 50 MP_30_CAR or MP_30_CD46.

The presence of CAR or CD46 molecules at the surface of MP_30_ and MP_100_ was confirmed by immunoelectron microscopy (immuno-EM), using anti-CAR or anti-CD46 mouse monoclonal antibody followed by a secondary 10 nm-gold-labeled anti-mouse IgG. The proportion of MP_30_ or MP_100_ associated with anti-CD46-bound or anti-CAR-bound colloidal gold grains was found to range between 2 to 3% (**Fig. 1A, B**). This value was consistent with the flow cytometry data, considering that MP_30_ and MP_100_ adsorbed on a solid support did not offer a full access to antibodies, compared to MPs in suspension.

### CD46 and CAR Molecules Displayed on MP_30_ were Functional as Adenoviral Receptors

**Figure 2:**
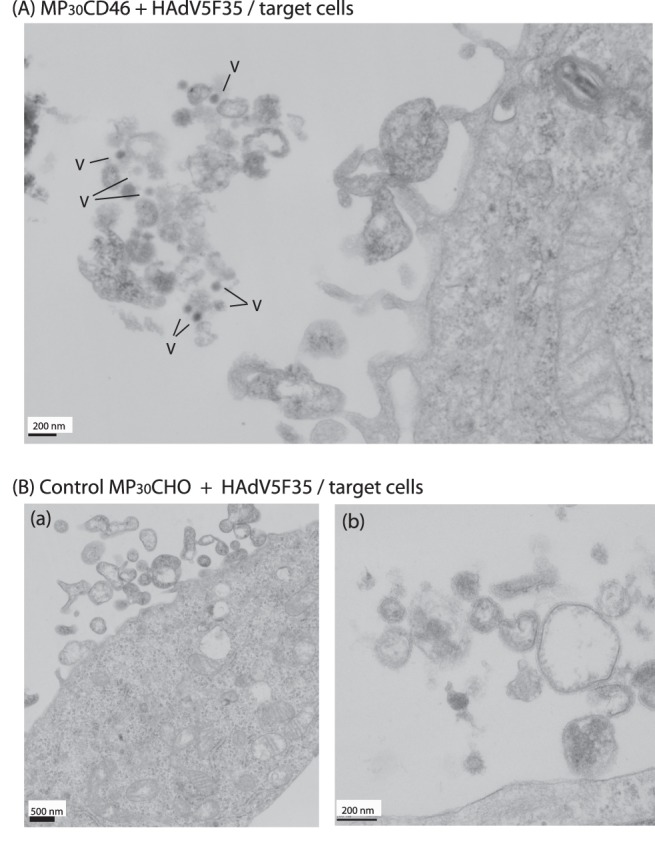
EM of target cells incubated with control MP_30_CHO or MP_30_CD46 in complex with HAdV5F35. (**A**), Ultrathin sections of target CHO cells incubated with MP_30_CD46-HAdV5F35 complexes. V, particles of HAdV5F35 vector. (**B**), Ultrathin sections of target CHO cells incubated with control MP_30_CHO from nontransduced CHO cells, mixed with HAdV5F35. Sections are shown at low (a) and high (b) magnifications. Note the absence of vector particles associated with control MP_30_CHO.

To assess the functionality of CAR and CD46 molecules on MP_30_ as adenoviral receptors, samples of MP_30_CAR and MP_30_CD46 were incubated with HAdV5-GFP or HAdV5F35-GFP vectors for 2 h at 37°C, using equal numbers of MPs and vector particles. The samples were then layered over a 20%-sucrose cushion, and centrifuged at 30,000×*g* for 2 h. The material which pelleted through the cushion was fixed and embedded, and ultrathin sections were processed for observation under the electron microscope (EM). Numerous complexes of MP_30_-viral vector particles were observed in the 30,000×*g*-pelletable fraction, as exemplified with MP_30_CD46 and HAdV5F35-GFP (**Fig. 1C**). These results confirmed that CD46 and CAR molecules displayed on MP_30_ were functional as cellular receptors for their specific adenoviruses. MP_30_CHO from unmodified, parental CHO cells were used as negative control. MP_30_CHO and MP_30_CD46 were incubated with aliquots of HAdV5F35-GFP vector, as above, and the mixtures added to monolayers of target CHO cells. Samples were harvested after 2 h at 37°C, and processed for EM. Particles of adenoviral vector were observed in complex with MP_30_CD46 at the surface of the target cells (**Fig. 2A**), whereas no vector particle in association with control MP_30_CHO was observed (**Fig. 2B**).

### MP-mediated Transfer of CAR and CD46 to Target Cells

#### (i) Transfer efficiency

To evaluate this parameter, aliquots of MP_30_CAR and MP_30_CD46 were added to CHO cell monolayers (10^5^ cells per well; 5 MP_30_/cell), and incubated for 2 h at 37°C. The supernatant was then removed and replaced by prewarmed complete medium. The cells were further incubated at 37°C, and cell samples were harvested at different time intervals, from 6 h to 10 days. The presence of CAR and CD46 molecules on the surface of recipient cells was determined by flow cytometry. The percentage of CAR- or CD46-positive cells was found to be approximately 3% at 6 h posttransfer (pt), 6–7% at 24–48 h, and 15–18% after 72 h. The plateau at about 15–18% was maintained until day-5, and progressively declined after one week (not shown).

#### (ii) Functionality of CAR and CD46 in MP-recipient cells (MP dose-dependence)

**Figure 3:**
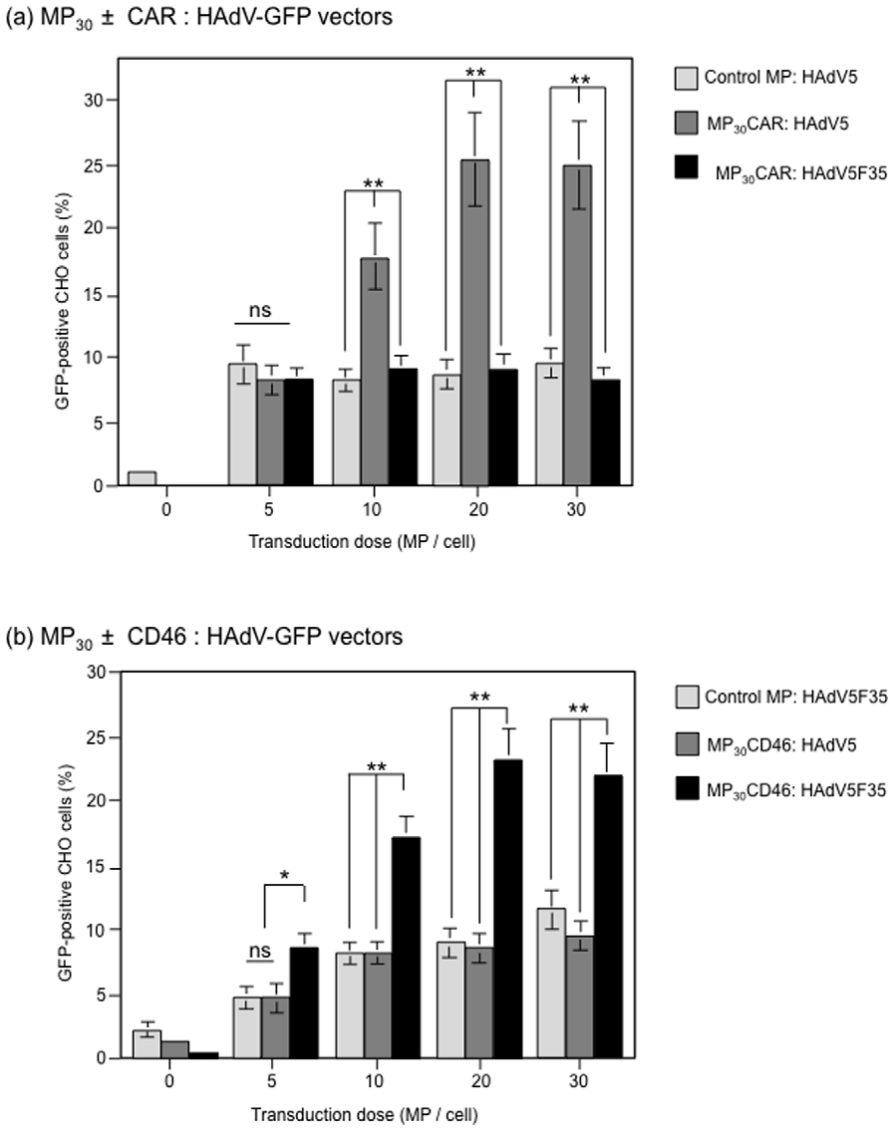
MP_30_-mediated transfer and functionality of (a) CAR, or (b) CD46 as adenoviral receptors in target cells. Aliquots of CHO cells (target cells) were incubated with MP_30_CAR (a) or MP_30_CD46 (b) at different MP doses per cell, as indicated in the *x*-axis. At 72 h after MP_30_-cell interaction, cells were infected with HAdV5-GFP or HAdV5F35-GFP vector, at the same MOI (500 vp/cell). The degree of CHO permissiveness to HAdV5-GFP or HAdV5F35-GFP vector was evaluated by flow cytometry analysis of the intracellular GFP signal. In (**a**), HAdV5F35-GFP, which does not recognize CAR as cellular receptor, was used as the negative control. In (**b**), HAdV5-GFP, which does not recognize CD46 as cellular receptor, was used as the negative control. MP_30_ from nontransduced CHO cells (Control MP) served as the negative controls in both panels.

To determine whether CAR and CD46 were functional as adenoviral receptors in MP-transduced cells, CHO cells were incubated with increasing doses of MP_30_CAR or MP_30_CD46, ranging from 0 to 30 MP_30_/cell, and assayed at 48 h pt for their permissiveness to HAdV5-GFP and HAdV5F35-GFP, respectively. HAdV5-GFP and HAdV5F35-GFP were used at a constant MOI of 500 vp/cell. As negative control, MP_30_CHO from parental CHO cells were used at the same doses. With MP_30_CHO, or at low doses of MP_30_CAR and MP_30_CD46 (≤5 MP_30_/cell), 2 to 10% cells were found to become GFP-positive (**Fig. 3**), which corresponded to the background of infection of CHO cells by HAdV5 and HAdV5F35 via other cell surface molecules, such as heparan sulfate glucosaminoglycans, which act as alternative receptors for HAdV5 and HAdV5F35 [43], [64]. However, CHO cells pre-incubated with MP_30_CAR or MP_30_CD46, and infected with HAdV5-GFP and HAdV5F35-GFP, respectively, showed a 2- to 3-fold increase in GFP-positive cells. This significant increase in permissiveness to the adenovirus vectors was MP dose-dependent (**Fig. 3**). These results indicated that MP_30_CAR and MP_30_CD46 were capable of transfering the CAR and CD46 molecules to CHO target cells.

#### (iii) Receptor specificity in MP-recipient cells

As additional controls, we assessed the specificity of vector-receptor recognition, by infecting MP_30_CD46-transduced cells with HAdV5-GFP, and *vice versa*, MP_30_CAR-transduced cells with HAdV5F35-GFP. Only a background GFP signal was detected with these two heterotypic pairs, demonstrating the specificity of the newly acquired CAR and CD46 receptor molecules towards their respective adenoviral vectors **(** **Fig. 3** **)**.

#### (iv) Functionality of CD46 as complement regulatory factor in MP-recipient cells

**Figure 4:**
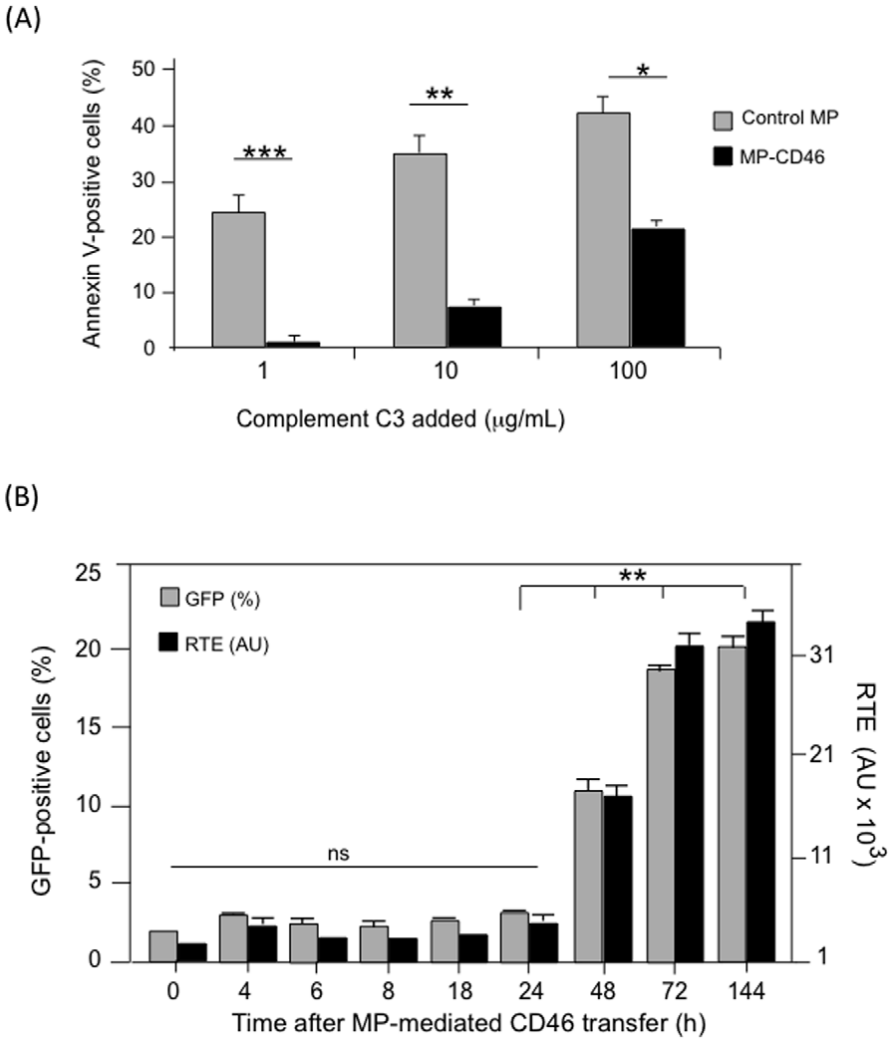
Functionality of exogenous CD46 in MP_30_CD46-transduced CHO cells. (A), CD46 as complement C3 regulator. CHO cells were harvested at 48 h posttransfer, and the CD46-induced protection against complement C3-mediated cell apoptosis was assayed by the percentage of Annexin V-positive cells determined by flow cytometry at increasing doses of complement C3. **(B), Kinetics of the gain of adenoviral receptor function by MP_30_CD46-transduced CHO cells.** CHO cells were harvested at different times after MP_30_CD46-transfer, and cell permissiveness to the HAdV5F35-GFP vector was assessed by infection with HAdV5F35-GFP at MOI 500. Cells were analyzed for GFP signal at 48 h postinfection. The degree of permissiveness to the vector was expressed as the percentage of GFP-positive cells (left *y*-axis), and the relative transduction efficiency (RTE; right *y*-axis). The RTE, in arbitrary units (AU), was given using the formula = (percentage of GFP-positive cells) x (MFI; mean fluorescence intensity).

The antiapoptotic activity of CD46 transferred to CHO cells was analyzed in CHO cells interacted with MP_30_CHO or with MP_30_CD46, then incubated with increased doses of complement fraction C3. A significant effect of protection against complement lysis was observed in MP_30_CD46-transduced CHO cells, by comparison to control cells (**Fig. 4A**).

### Influence of VSV-G Incorporation on MP_30_-mediated Transfer of CAR or CD46

The following experiments were designed to determine whether the transfer of CAR or/and CD46 to target CHO cells would be improved by the incorporation of VSV-G, the envelope glycoprotein G of vesicular stomatitis virus, into the MP envelope. We found that baculovector-mediated expression of VSV-G in CHO-CAR and CHO-CD46 cells did not enhance the production of CAR- or CD46-pseudotyped MPs (data not shown). Likewise, coincorporation of VSV-G with CAR or CD46 into MP_30_ did not significantly increase the degree of permissiveness of CHO cells to HAdV5-GFP or HAdV5F35-GFP after transduction by MP_30_CAR-VSV-G or MP_30_CD46-VSV-G (**Fig. S2**). This implied that VSV-G was not a facilitator of MP_30_ uptake by the target CHO cells, and VSV-G was not included in the following experiments.

### Time-course Analysis of the Acquisition of New Functionality by MP_30_-recipient Cells

The next issue was to determine at which time after MP_30_CD46-cell interaction the CHO target cells acquired the adenovirus receptor function carried by CD46. CHO cells were incubated with a constant dose of MP_30_CD46 (20 MP_30_/cell), and at different times after MP_30_-cell interaction, the cells were incubated with HAdV5F35-GFP vector at constant vector MOI (500 vp/cell) for 2 h at 37°C. At 48 h post-infection (pi), the permissiveness of the cells to HAdV5F35-GFP was determined by flow cytometry analysis. A progressive increase in adenoviral permissiveness was observed, with 10% GFP-positive cells at 48 h pi, reaching a plateau of 20–25% GFP-positive cells at 72–144 h (**Fig. 4B**). Similar results were obtained with MP_30_CAR (not shown). The kinetic data implied that a larger proportion of cells became permissive to adenovirus with time, and suggested the contribution of additional CD46 molecules. This was likely due to newly synthesized CD46 from CD46-encoding mRNA transferred by MP_30_CD46 to the target cells, as demonstrated below for MP-mediated CFTR transfer.

### Generation of CHO Cells Stably Expressing GFP-CFTR

**Figure 5:**
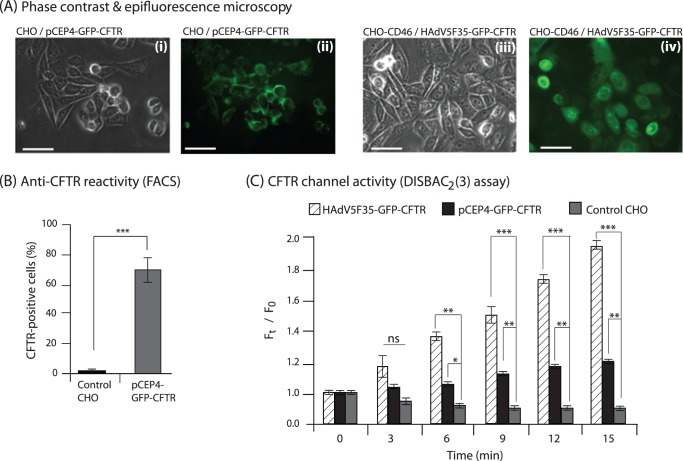
Expression, immunoreactivity and functionality of GFP-CFTR protein expressed from an episomal plasmid in CHO cells (MP-producer cells). (**A**), CHO cells harboring the GFP-CFTR-encoding pCEP4 episomal plasmid, were examined by phase-contrast (**i**) and epifluorescence microscopy (**ii**) of GFP-tagged CFTR protein. Positive controls, consisting of CHO cells transduced by the adenoviral vector HAdV5-GFP-CFTR were similarly examined by phase-contrast (**iii**) and epifluorescence microscopy (**iv**). Scale bars, 20 µm. (**B**), Surface expression and correct orientation of the GFP-tagged CFTR protein in pCEP4-GFP-CFTR harboring CHO cells, evaluated by flow cytometry using monoclonal antibody against the first extracellular loop of the CFTR ectodomain. (**C**), Chloride channel activity of CFTR in negative control CHO cells (dotted bars), positive control HAdV5F35-GFP-CFTR-transduced CHO-CD46 cells (hatched bars), and pCEP4-GFP-CFTR harboring CHO cells (black bars), evaluated using the fluorescent probe DiSBAC_2_(3). The time-course analysis of DiSBAC_2_(3) fluorescence changes in the presence of a cocktail of CFTR activators was monitored in regions of the cell monolayers corresponding to 20–30 cells. Bars represent the mean fluorescence value for each field of 20–30 cells ± SEM. Symbols: *, *p*<0.05; **, *p*<0.01; ns, not significant.

A CHO cell line stably expressing GFP-fused human CFTR was generated (CHO-GFP-CFTR) using the pCEP_4_-GFP-CFTR episomal plasmid. The pCEP_4_-GFP-CFTR-directed expression of the GFP-CFTR fusion protein in these cells showed localisation of GFP signal in the cytoplasm, perinuclear and submembrane regions (**Fig. 5A** **i, ii;** and **Fig. S3A**). A similar fluorescence pattern was observed in CHO-CD46 cells transduced by the HAdV5F35-GFP-CFTR vector which were used as positive control (**Fig. 5A** **iii, iv**). In CHO-GFP-CFTR cells, the CFTR molecules were correctly oriented in the plasma membrane, as shown by the labeling of nonpermeabilized CHO-GFP-CFTR cells with an anti-CFTR monoclonal antibody directed against the first extracellular loop of the CFTR ectodomain (**Fig. 5B**).

The chloride channel function in CHO-GFP-CFTR cells was investigated using the voltage-sensitive probe DiSBAC_2_(3). The fluorescent signal of DiSBAC_2_(3) has been shown to vary with changes of the membrane potential induced by the cAMP/low-chloride-mediated activation of the CFTR [65]. Parental CHO cells were used as negative controls, and CHO-CD46 cells transduced by the HAdV5F35-GFP-CFTR vector were used as positive controls. A positive fluorescent signal was observed in CHO-GFP-CFTR cells in response to the addition of the cAMP-based cocktail of CFTR activators, demonstrating the functionality of the exogenous GFP-CFTR molecules as chloride channels (**Fig. 5C**). HAdV5F35-GFP-CFTR-transduced cells used as positive control showed a strong enhancement of the fluorescent signal in the presence of CFTR activators (**Fig. 5C**), consistent with the high transduction efficiency of CHO-CD46 by HAdV5F35 [55], [66]. CHO-GFP-CFTR cells were then tested for the production of MPs carrying GFP-CFTR (MP-GFP-CFTR) and the MP-mediated delivery of CFTR to target cells.

### Isolation and Recovery of MP-GFP-CFTR

MP_30_GFP-CFTR and MP_100_GFP-CFTR were recovered from the CHO-GFP-CFTR cell culture medium by the ultracentrifugation procedure mentioned above and depicted in **Fig. S1**. The presence of the GFP-tag allowed the direct tracking of GFP-CFTR molecules in the different cell compartments, such as the vesicular compartment and plasma membrane [55], [66]. It also allowed the detection of GFP-CFTR incorporated in MPs derived from GFP-CFTR-expressing cells. The recovery of MP_30_GFP-CFTR and MP_100_GFP-CFTR ranged from 1×10^7^ to 5×10^7^ per 10^7^ CHO-GFP-CFTR cells. Flow cytometry analysis indicated that the GFP-CFTR signal was detected in both MP_30_ and MP_100_ populations, although in significantly higher amounts in MP_30_ compared to MP_100_ : 10.3±3.3% GFP-CFTR-positive MP_30_ (m ± SEM, *n* = 3), *versus* 3.6±3.3% for MP_100_. The presence of CFTR in both MP populations was not surprising, considering the trafficking of CFTR molecules between intracellular membranal compartments and the cell surface.

### Fractionation of MP_30_GFP-CFTR and MP_100_GFP-CFTR Populations and Distribution of GFP-CFTR between MP Subclasses

To obtain the maximum efficiency of GFP-CFTR transfer to target cells, it was important to determine whether a particular MP fraction could be enriched in GFP-CFTR. To this aim, the MP_30_ and MP_100_ populations from CHO-GFP-CFTR cells were further fractionated into subpopulations differing in density, using ultracentrifugation in isopycnic gradients [67]. Three subpopulations, corresponding to three density classes, were obtained for each MP population (**Fig. S1**): (i) MPs of low density (MP_30_LD and MP_100_LD) with a mean density (ρ_m_) of 1.10; (ii) MPs of intermediate density (MP_30_ID and MP_100_ID; ρ_m_ = 1.16); and (iii) MPs of high density (MP_30_HD and MP_100_HD; ρ_m_ = 1.21). Each subclass of MP_30_ and MP_100_ populations was then assayed for the presence of GFP-CFTR by flow cytometry analysis. The GFP-CFTR protein was detected in all MP subclasses, with the highest proportion of GFP-CFTR-positive MPs found in the high-density compared to low-density subclasses: 14.5% for MP_30_HD versus 6.2% for MP_30_LD, and 8.3% for MP_100_HD versus 1.4% for MP_100_LD (**Table 1**). This data suggested that CFTR glycoprotein was associated with the higher density MPs.

**Table 1:** Proportion of GFP-positive MPs in the different MP subclasses, and GFP-CFTR mRNA content (a).

Density subclass	GFP(+) MP_30_ (% total MP)	GFP(+) MP_100_ (% total MP)	GFP-CFTR RNA in MP_30_ (b)	GFP-CFTR RNA in MP_100_ (b)
LD (ρ_m_ = 1.10)	6.2±2.5	1.4±0.4	82.5±14.3	97.7±77.0
ID (ρ_m_ = 1.16)	10.4±1.7	1.2±1.2	61.6±35.2	44.0±32.3
HD (ρ_m_ = 1.21)	14.5±3.2	8.3±2.1	51.3±30.7	60.5±22.5

(a) The proportion of GFP-positive MPs was determined by flow cytometry, and the GFP-CFTR mRNA content by qRT-PCR analysis. Data shown in the Table are mean values (m) ± SEM (*n* = 3).

(b) The values obtained after RT-PCR amplification of a RNA fragment of 196 nt in length overlapping the GFP C-terminal and CFTR N-terminal sequences were expressed as ng RNA per 10^7^ MP_30_ or MP_100_.

### Kinetics of MP-mediated Transfer of GFP-CFTR to Target Cells

**Figure 6:**
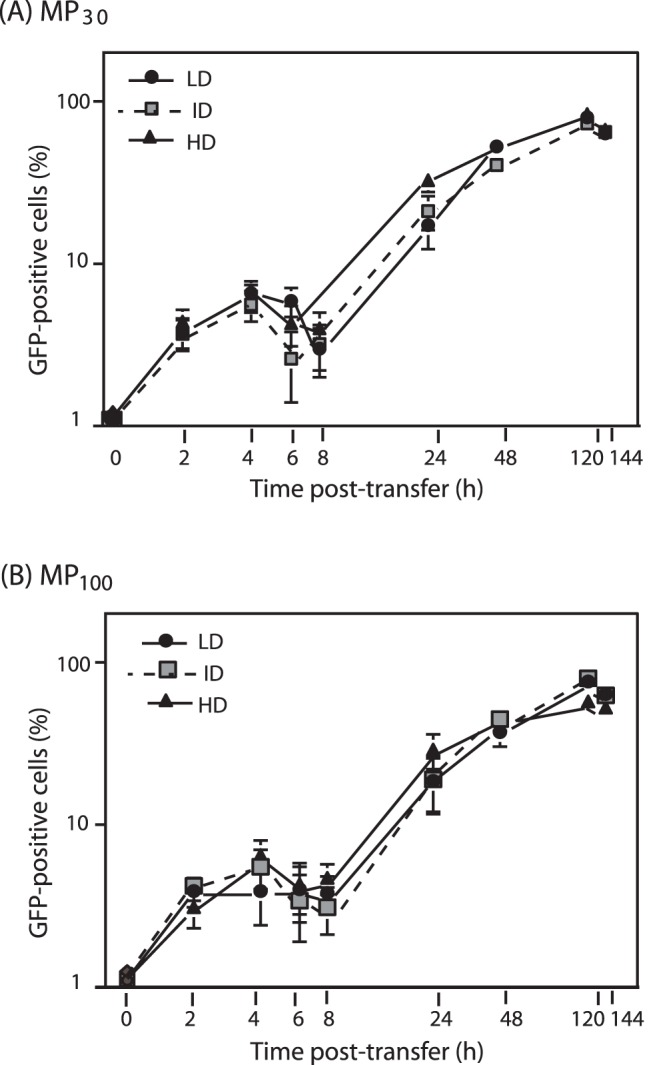
Time-course expression of GFP-CFTR in MP-transduced CHO cells. (**A**), (**B**), GFP-positive cells (expressed as %) were determined by flow cytometry analysis of MP_30_-transduced (**A**) and MP_100_-transduced cells (**B**), harvested at different times post-transfer (pt). Bars represent mean values ± SEM (*n* = 3).

The following experiments were designed to determine the capacity of each subpopulation of MPs to transfer GFP-CFTR to target CHO cells. The MP_30_ (LD, ID and HD), and MP_100_ (LD, ID and HD) subclasses were incubated with CHO cells at a ratio of 5 MPs/cell for 2 h at 37°C. Inocula were removed, and monolayers were postincubated with fresh, prewarmed medium at 37°C for 6 days. At different time-points, cell samples were examined by fluorescence microscopy and flow cytometry for qualitative and quantitative analyses of the GFP signal, respectively. With the MP_30_ population, the GFP profiles did not differ significantly for the three subclasses. At 2 h pt, 3.8 to 4.4% CHO cells were GFP-positive, increasing to 5.5–6.6% at 4 h pt, followed by a slight decrease at 6–8 h pt (**Fig. 6A**). At 8 h pt, the proportion of GFP-positive cells increased progressively for the three MP_30_ subclasses, to attain a value of 73–83% GFP-positive cells 5 days pt (**Fig. 6A**). A similar kinetics of appearance of GFP-positivity was observed with the three MP_100_ subclasses: a discrete peak was detected at 4 h pt, followed by a slight decrease at 6–8 h pt, and a progressive increase to 57–76% GFP-positive cells after 5 days (**Fig. 6B**).

**Figure 7:**
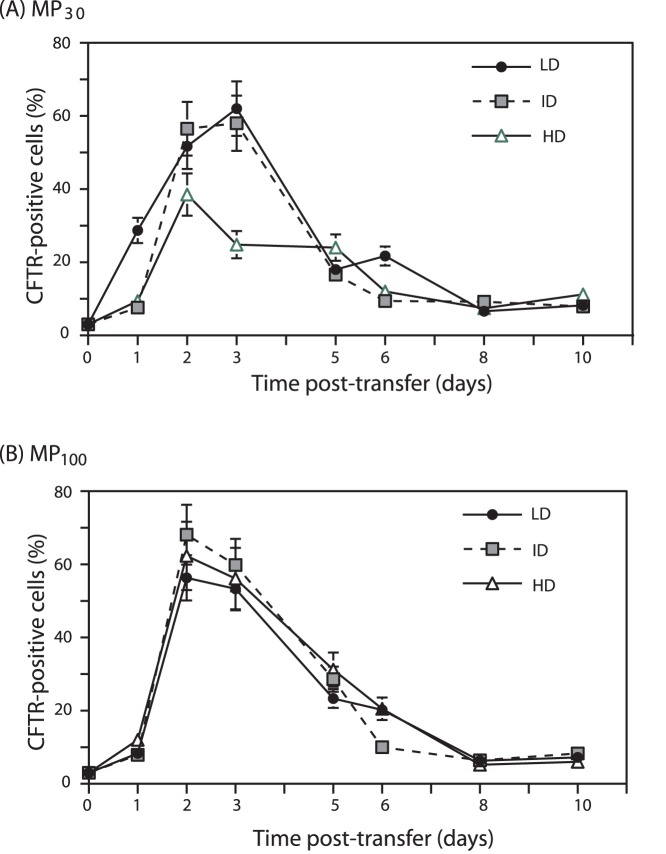
Time-course expression of GFP-CFTR glycoprotein at the surface of MP-transduced CHO cells. Cells harvested at different times after MP-cell transfer were analyzed by flow cytometry for the immunoreactivity of the first N-terminal loop of the CFTR ectodomain with anti-CFTR monoclonal antibody. (**A**), MP_30_-transduced CHO cells. (**B**), MP_100_-transduced CHO cells. Bars represent mean values ± SEM (*n* = 3).

The cell surface expression and proper membrane insertion of GFP-CFTR was investigated by flow cytometry, using the anti-CFTR ectodomain antibody as above. The maximal CFTR display at the cell surface was observed at 2–3 days pt, with 50–60% CFTR-positive cells after MP_30_-mediated transduction (**Fig. 7A**). Similar levels (60–70% CFTR-positive cells) were observed after MP_100_-mediated transduction (**Fig. 7B**). The presence of CFTR at the cell surface progressively declined after day-4 (**Fig. 7A, B**).

Interestingly, there was a slight, but significant difference in profile between the GFP signal and the CFTR ectodomain immunoreactivity. The proportion of GFP-positive cells remained as a plateau after 6 days (refer to Fig. 6 A, B) whereas the display of CFTR at the cell surface decreased after day-4 (refer to Fig. 7). Likewise, MP_30_- and MP_100_-recipient cells examined at day-5 pt by confocal microscopy showed that the majority of the GFP-CFTR signal appeared as cytoplasmic dots and speckles corresponding to vesicular compartment(s), and in relatively low proportion at the plasma membrane (**Fig. S3B**).

### Occurrence of GFP-CFTR-encoding mRNA in MPs and MP-recipient Cells

**Figure 8:**
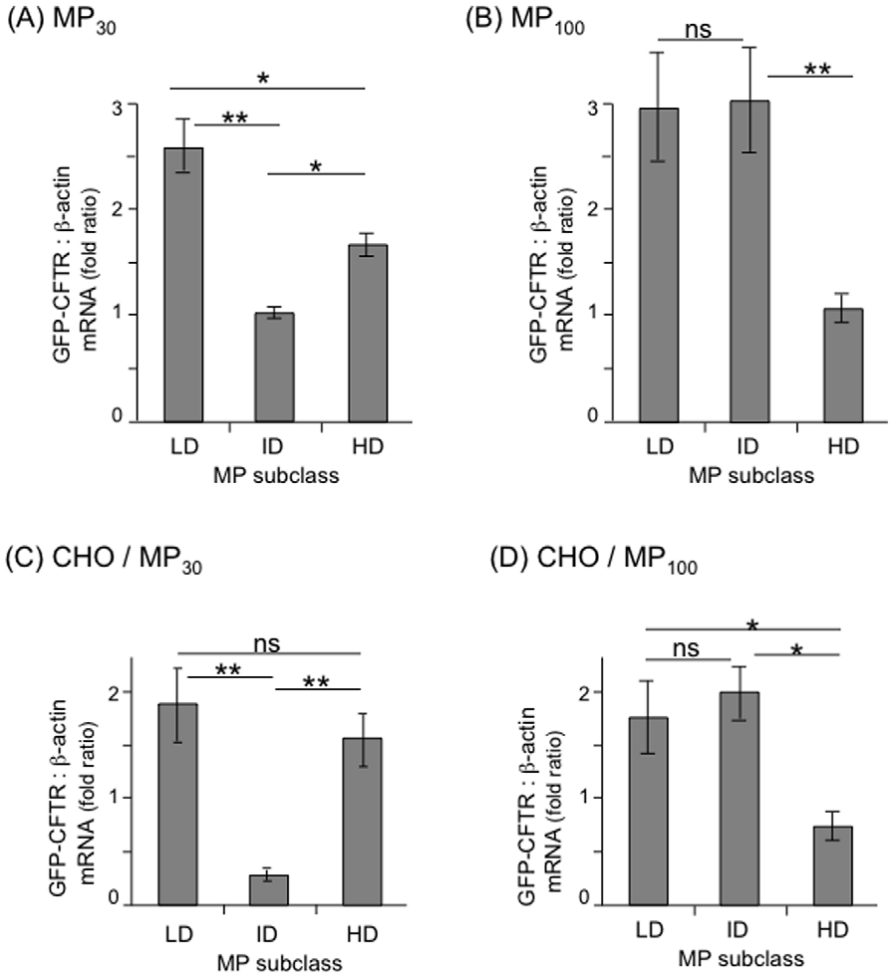
Occurrence of GFP-CFTR-encoding mRNA in MPs and MP-transduced CHO cells. Bar graph representation of qRT-PCR assays of MP_30_ (**A**), MP_100_ (**B**), MP_30_-transduced CHO cells (**C**), and MP_100_-transduced CHO cells (**D**). Data shown in the graphs are mean values (m) ± SEM (*n* = 3). Symbols: *, *p*<0.05; **, *p*<0.01; ***, *p*<0.001; ns, not significant.

The kinetics of appearance of GFP-positive cells shown in Fig.5 suggested an early (4 h pt) and a late phase (3 days pt) of MP-mediated GFP-CFTR transfer. We hypothesized that at early times after interaction of MP_30_ and MP_100_ with target cells, membrane-inserted GFP-CFTR glycoprotein molecules were transferred to a small number of target cells (less than 10%), following a pathway of MP-cell binding, endocytosis and membrane fusion. At late times, the GFP-CFTR protein detected in the MP-recipient cells was likely due to the neosynthesis of GFP-CFTR from MP-embarked GFP-CFTR-encoding mRNA molecules. To test this hypothesis, total RNA were extracted from the different MP_30_ and MP_100_ subclasses, and analysed for the presence of GFP-CFTR-encoding mRNA (mRNA*^gfp-cftr^*), using quantitative RT-PCR and specific primers overlapping the junction of the GFP C-terminal and CFTR N-terminal coding sequences [55], [66]. All MP subclasses were found to contain comparable amounts of mRNA*^gfp-cftr^* (**Table 1**). However, when the values of the MP content of mRNA*^gfp-cftr^* were expressed as the ratio to β-actin mRNA content used as endogenous control, significant differences in composition were observed between the MP subclasses (**Fig. 8A, B**).

Similarly, total RNA were extracted from MP-recipient cells incubated with MP_30_ (LD, ID and HD), and MP_100_ (LD, ID and HD) at day-5 pt, and qRT-PCR was carried out as above, using β-actin mRNA as internal control. As positive controls, CHO-CD46 cells transduced by HAdV5F35-GFP-CFTR were processed for RNA extraction and qRT-PCR assays. We found that the recipient cells incubated with all the different subclasses of of MP_30_ and MP_100_ contained mRNA*^gfp-cftr^*, with a ratio to β-actin content varying from 0.5- to 2-fold (**Fig. 8C, D**). Interestingly, the variations in the cellular content of mRNA*^gfp-cftr^* reflected the respective contents of the different MP subclasses. The observation that almost 80% of the cells expressed GFP-CFTR protein at late times pt suggested that nearly all cells received mRNA*^gfp-cftr^* molecules when a bioactive MP-to-cell ratio of 5 MPs/cell was used.

The efficiency of transfer of mRNA*^gfp-cftr^* was calculated as follows. The average content of donor cells was found to be 4.4×10^8^ copies of mRNA*^gfp-cftr^* per µg total cellular RNA extracted, with values ranging from 2.2×10^8^ to 6.6×10^8^ copies. The average content of MP-recipient cells incubated with MP_100_-GFP-CFTR was 3.5×10^7^ copies of mRNA*^gfp-cftr^* at 48 h after transfer using 5 MPs/cell, with values ranging from 1.7×10^7^ to 5.3×10^7^ copies per µg total cellular RNA. We estimated the transfer efficiency as the ratio 3.5×10^7^ : 4.4×10^8^ ≈ 8%.

To eliminate the possibility of DNA transfer via MPs, CHO cells were harvested at 48 h after incubation with each of the different subclasses of MP_30_ and MP_100_, the DNA extracted and probed for the episomal plasmid pCEP_4_-GFP-CFTR, using PCR amplification of a 196 nt fragment overlapping the *GFP*-3' and *CFTR*-5' sequences [66]. No specific DNA fragment was found at this position in all cell samples tested (**Fig. S4**).

### Functionality of Exogenous CFTR as Chloride Channel in MP-recipient Cells

**Figure 9:**
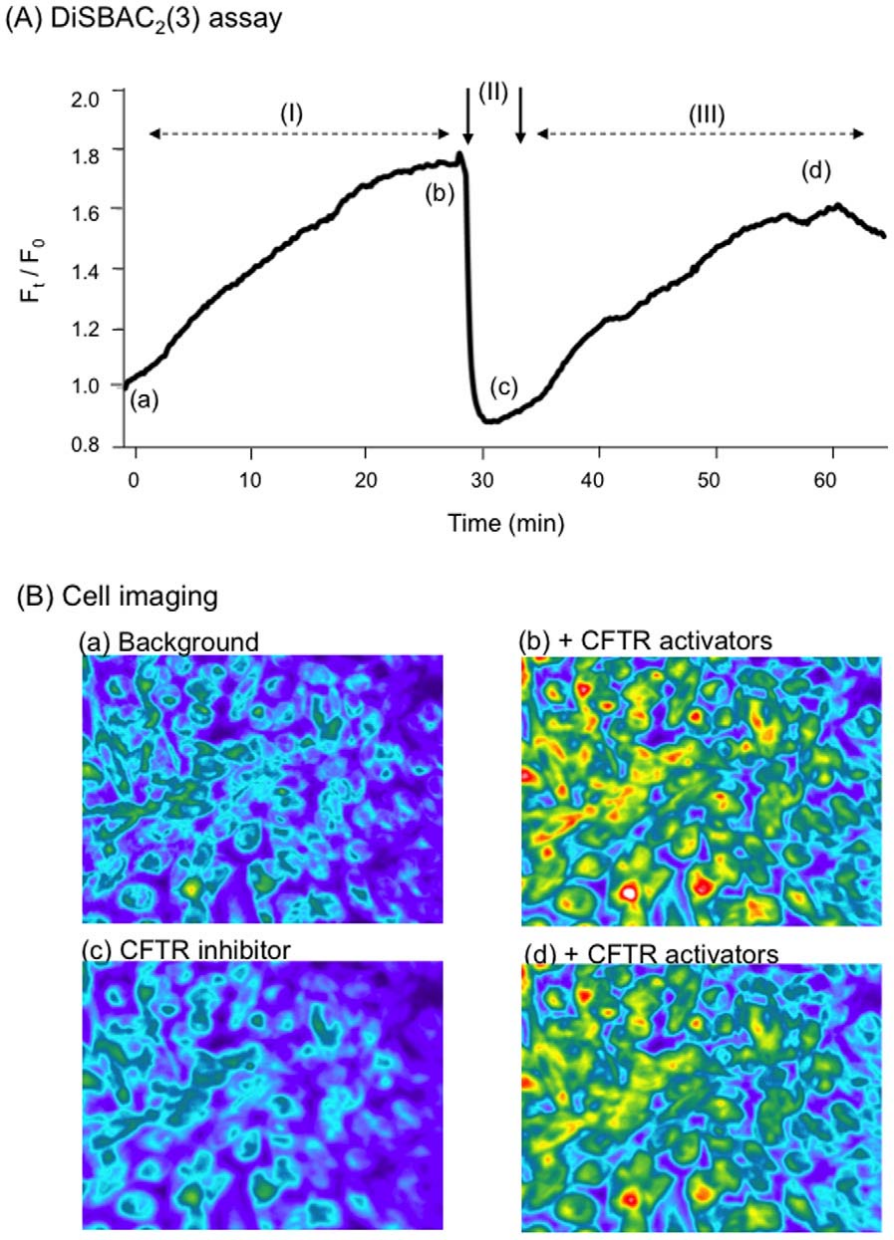
CFTR channel activity **in MP-transduced CHO recipient cells. (A),** ***Evolution of the DiSBAC_2_(3) fluorescence signal***. The time-course analysis of DiSBAC_2_(3) fluorescence changes was monitored in regions of the cell monolayer corresponding to 20–30 cells, taken at 72 h post-transfer, a time point corresponding to the maximal immunoreactivity of CFTR glycoprotein at the cell surface. Changes in the fluorescent signal were expressed as F_t_/F_0_ ratio values, in which F_t_ and F_0_ were the fluorescence values at the times *t* and *t*_0_, respectively, and *t*_0_ the time when the cAMP-containing, CFTR-activating cocktail was added. The cocktail of CFTR activators was maintained throughout the experiment. The CFTR inhibitor GlyH-101 was added for 5 min (phase II, marked by two vertical arrows). Phase III shows reversibility of the CFTR block and recovery of the fluorescent signal. **(B),** ***Fluorescence microscopy***. Photographs of cell monolayers were taken at different time-points corresponding to the four successive phases, as indicated on the curve by the letters (a), (b), (c) and (d).

The next issue to address was whether target CHO cells incubated with MP-GFP-CFTR acquired the CFTR-associated chloride channel activity. CHO cells were taken 3 days after incubation of MP_30_-GFP-CFTR or MP_100_-GFP-CFTR, the time at which a maximum number of cells were observed to be GFP- and CFTR-positive (refer to Fig. 6 and 7). The changes of the DiSBAC_2_(3) fluorescent signal in MP-transduced cells in response to the CFTR activator and inhibitor were monitored by quantitative fluorescence microscopy (**Fig. 9**). As exemplified with MP_100_-GFP-CFTR, the fluorescent signal increased progressively in the presence of CFTR activators, and rapidly decreased to background levels upon the addition of the CFTR inhibitor GlyH-101 (**Fig. 9**). This effect was reversible, as the fluorescence recovered progressively with the removal of GlyH-101 from the cAMP-containing superfusion medium (**Fig. 9**). Thus, the target CHO cells showed a gain in biological function, i.e. the chloride channel activity associated with acquired CFTR molecules.

## Discussion

The current trends in biotherapy comprise the development of stem cell technology in combination with *ex vivo* gene therapy, the improvement of synthetic vectors, or the design of immunologically stealthy viral vectors, or safer viral integrative vectors with controlled integration sites in host cells. The present study explored an alternative strategy to transfer therapeutic biomolecules to target cells, using MPs as conveyors. MPs have been reported to successfully mediate the transfer of surface molecules, such as receptors and extracellular matrix proteases, and also intracellular components, including proteins, lipids and RNA molecules of different types [1]–[3], [27].

In the present study, the cellular model for MP production and uptake was the Chinese Hamster Ovarian (CHO) cell line, used as MP-producer and MP-recipient cells. This choice was based on the following considerations. (i) CHO cells did not express orthologs of the human CAR, CD46 and CFTR glycoproteins, which made possible the assessment of the capability of MPs to transfer the CAR-, CD46- and CFTR-specified function(s) to these target cells. (ii) Previous experiments of expression of human CFTR, CAR and CD46 in CHO cells have shown the functionality of these proteins and their localisation at the plasma membrane [44], [48], [63], [68]. (iii) A homologous cell system, with the maximal degree of compatibility between MP-donor and MP-recipient cells in terms of membrane composition, would optimize the cell-to-cell transfer of biological material.

Three types of human cell surface molecules with different degrees of complexity were used as prototype bioactive glycoproteins in our study: (i) CAR and (ii) CD46, which are both monospanins and act as high affinity receptors for human adenovirus serotypes 5 and 35, respectively; and (iii) CFTR, a complex transmembrane glycoprotein which functions as a chloride channel. The MP-donor cells were CHO cell lines which constitutively expressed CAR, CD46, or CFTR. MP-target cells were naive CHO cells, which lacked CAR and CD46 and did not express the equivalent of the human CFTR glycoprotein. In all the three cases, the MP-donor cells were grown in the absence of chemical or biological stress, to avoid the occurrence of apoptotic factors within MPs and the possible transfer of stress signals to recipient cells. The populations of MPs issued from CAR-, CD46- or CFTR-expressing CHO cells were separated according to their size by velocity ultracentrifugation, yielding MP_30_ and MP_100_ fractions.

We found that it was possible to transfer CAR and CD46 to target CHO cells using MPs as vehicles, resulting in the acquisition of new biological functions by the target cells. CHO cells incubated with MP_30_CAR and MP_30_CD46 became permissive to HAdV5 and AdV5F35 infection, respectively. In addition, cells incubated with MP_30_CD46 acquired resistance to complement C3-induced apoptosis. The MP-mediated transfer of CD46 might have potential therapeutic applications in the control of allograft rejection, as well as in gene therapy and/or vaccinology using HAdV35-based vectors. The MP-mediated transfer of CAR might be used to confer viral permissiveness to adenovirus-refractory cells, such as tumor cells towards HAdV5-based oncolytic vectors. The viral glycoprotein VSV-G, which has a high fusiogenic activity, has been advantageously used to augment the efficacy of membrane fusion between virus (or VLP) and target cells [35], [37]–[39], [69]–[71]. In the present study however, coincorporation of VSV-G at the surface of MP_30_CAR or MP_30_CD46 did not improve the transfer efficiency of CAR or CD46 molecules to target cells.

In the case of CFTR, MPs were isolated from the culture medium of CHO cells which were engineered to constitutively express GFP-CFTR. MP_30_GFP-CFTR and MP_100_GFP-CFTR obtained by velocity ultracentrifugation were further fractionated into three density subclasses: MPs of low, intermediate and high density, respectively. All MP subclasses incorporated significant levels of GFP-CFTR protein as well as mRNA*^gfp-cftr^*, with no significant variations between the different subclasses. Likewise, all MP subclasses were competent in transferring GFP-CFTR to target CHO cells. The kinetics of appearance of the GFP signal in these cells showed two separate peaks, which suggested an early (4–6 h) and late phase (3 days) of protein transfer after MP-cell interaction. The early GFP signal detected in target cells likely resulted from the transfer of GFP-CFTR protein via different mechanisms described in the literature [1]–[4], such as the direct fusion between MP membrane and target cell plasma membrane, endocytosis of the MPs and recycling of GFP-CFTR to the cell surface, or the coexistence of both mechanisms. Interestingly, the incorrect insertion and orientation of a protein in the plasma membrane of recipient cells have been reported [72]. In the present study, the GFP-CFTR was inserted in the plasma membrane of target cells in the correct in-out orientation, as validated by an antibody recognising an extracellular loop of the CFTR.

At the late phase after MP transfer, almost 80% of the target cells became positive for GFP-CFTR, due to GFP-CFTR molecules issued from *de novo* protein synthesis directed by exogenous mRNA*^gfp-cftr^* (free or/and polysomal) transferred over by MP_30_ or MP_100_. Attempts to block the protein synthesis machinery in MP-recipient CHO cells using cycloheximide (CH) were performed to further explore this mechanism. However, the results were inconclusive, due to the unexpected high cytotoxicity of CH towards GFP-CFTR-expressing CHO cells, when treated with the protein synthesis inhibitory doses usually applied to human cells (10 to 20 µg/mL; [73]). Of note, the half-life of the *CFTR* gene products in human cells has been determined to be ∼48 h for the CFTR glycoprotein [74], [75], and ≥20 h for the mRNA*^cftr^* [76].

MPs have been shown to carry and transfer mRNAs and microRNAs to target cells, as a general mechanism of intercellular communication [3], [5], [7], [11], [27], [29]. MP-delivered RNA has been termed shuttle RNA ( [11]), and has been found to be functional in the new cellular context [7], [29], [77]–[80]. Consistent with the results of these previous studies, our experimental data showed that our shuttling mRNA*^gfp-cftr^* was functional, and that neosynthesized GFP-CFTR glycoproteins were addressed to the cell surface of target cells, correctly inserted into their plasma membrane, and metabolically active in anion transport. Using a fluorescent voltage sensitive DISBAC_3_(2) probe assay, the CFTR-associated chloride channel activity was detected in MP-recipient cells at the late phase (day-3), when the translational machinery from the exogenous mRNA*^gfp-cftr^* became fully operational. The chloride channel function was not detectable in MP-recipient cells at early times, possibly due to a threshold in the detection of this activity using the fluorescent probe.

Our observation that MP_30_ and MP_100_ produced by CHO-GFP-CFTR donor cells were equally capable of transferring mRNA*^gfp-cftr^* to target cells implied that both populations were competent for CFTR delivery. Fractionation of MP_30_ or MP_100_ by isopycnic ultracentrifugation did not result in any significant enrichment of a particular subclass in mRNA*^gfp-cftr^* molecules. However, in future studies using MPs issued from donor cells of human origin, fractionation of MPs specifically carrying mRNA*^cftr^* using MP sorting based on surface markers available in human cells, would be advantageous for a higher efficiency of CFTR delivery. Cell-derived MP_30_ or MP_100_ appeared therefore as promising biological vehicles of therapeutic mRNAs exerting their therapeutic function(s) indirectly, via their encoded proteins. In particular, in the case of cystic fibrosis, MPs have a potential application in nongenic, cell-to-cell autologous transfer of human mRNA*^cftr^* and the restoration of the normal chloride channel function in CFTR-deficient cells.

## Materials and Methods

### Cells and Plasmids

HEK-293 cells, obtained from the ATCC (Manassas, VA), were maintained as monolayers in Dulbecco's modified Eagle's medium (DMEM, Gibco-Invitrogen) supplemented with 10% fetal bovine serum (FBS, Gibco-Invitrogen), penicillin (100 U/ml) and streptomycin (100 µg/ml) at 37°C and 5% CO_2_. Chinese hamster ovarian cells (CHO-K1 or simply CHO) was obtained from the ATCC, CAR-expressing CHO cells (CHO-CAR) from Dr. J. Bergelson [44], and CD46-expressing CHO cells (CHO-CD46) from Dr. D. Gerlier [63].They were grown in Iscove’s medium supplemented with 10% FBS and 50 µg/ml gentamicin (Invitrogen). To generate the CHO cell line stably expressing GFP-CFTR, the GFP-CFTR gene was excised from the plasmid pGFP-CFTRwt described in a previous study [55], using *Nhe* I and *Sma* I digestion, and inserted into pCEP_4_ (Invitrogen, Life Technologies). This plasmid vector was designed for high-level, constitutive expression from the CMV promoter, and which contains the EBNA-1 gene for episomal maintenance in human cell lines. *Sfi* I-linearized and blunted pCEP4 was further digested with *Nhe* I. After purification by agarose gel electrophoresis, the *Nhe* I-cut pCEP4 was ligated with the GFP-CFTR-containing *Nhe* I-*Sma* I DNA fragment, generating the pCEP_4_-GFP-CFTR vector. CHO cell monolayers were transfected with pCEP_4_-GFP-CFTR, using Lipofectamine-2000 (Invitrogen), according to the manufacturer’s instructions. CHO cells harboring pCEP_4_-GFP-CFTR (referred to as CHO-GFP-CFTR) were selected using culture medium alpha-MEM with ribonucleosides (Invitrogen), supplemented with 5% FBS, penicillin (100 U/ml), streptomycin (100 µg/ml) and hygromycin-B (50 µg/mL). The episomal plasmid pCEP_4_-GFP-CFTR was found to be maintained in transfected cells for at least six months under hygromycin-B selection pressure.

### Isolation of MPs Carrying Human Glycoproteins CAR (MP-CAR), CD46 (MP-CD46) or GFP-CFTR (MP-GFP-CFTR) (Fig. S1)

#### (i) Velocity ultracentrifugation

MPs were recovered from the culture medium of nonstressed cells, consisting of control CHO (MP-CHO), CHO-CAR (MP-CAR), CHO-CD46 (MP-CD46 and CHO-GFP-CFTR (MP-GFP-CFTR). This was carried out by using a previously published centrifugation protocol [81] with the following modifications. Cells were seeded at 70–80% confluence, grown for 48 h to reach confluence (10^7^ cells), then maintained for an additional 24 h. Culture medium was clarified from cell debris by centrifugation for 2 min at 13,000×*g* and 4°C. This clarified supernatant (S0) was the source of MPs. S0 was centrifuged at 30,000×*g* and 4°C for 2 h. The pellet P1 was kept, and referred to as MP_30_. The supernatant (S1) was centrifuged at 100,000×*g* and 4°C for 2 h, to obtain pellet P2, referred to as MP_100_. Pellets P1 and P2 were resuspended in 200 µl phosphate buffered saline (PBS) with gentle mixing, for further analysis by flow cytometry, and stored at 4°C before use for MP-mediated protein transfer, or further MP fractionation.

#### (ii) Isopycnic ultracentrifugation

MP_30_ and MP_100_ were further fractionated by ultracentrifugation of flotation in isopycnic gradient, to separate the MP_30_ and MP_100_ fractions into subclasses differing by their apparent density [67]. Samples of MP_30_ or MP_100_ in PBS were placed at the bottom of a preformed linear sucrose-D_2_O gradient (10 ml total volume; 0.25 to 2.5 M sucrose). The 2.5 M sucrose solution was made in D_2_O buffered to pH 7.2 with NaOH, and the 0.25 M sucrose solution was made in 10 mM Tris-HCl, pH 7.2, 150 mM NaCl, 5.7 mM Na_2_EDTA. The gradients were centrifuged for 18 h at 100,000×*g* in a Beckman SW41 rotor. Fractions of 0.4 ml were collected from the top, and density measured by weighing 100 µl-aliquots, using a precision scale. Fractions were pooled according to their apparent density, and three pools were constituted : MPs of low density (MP_30_LD and MP_100_LD, respectively) corresponded to pooled fractions 1–7, with densities (ρ) ranging from 1.06 to 1.14 (mean ρ : ρ_m_ = 1.10), MPs of intermediate density (MP_30_ID and MP_100_ID) to fractions 8–14 (1.14≤ ρ ≤1.18; ρ_m_ = 1.16), MPs of high density (MP_30_HD and MP_100_HD) to fractions 15–21 (1.18≤ ρ ≤1.25; ρ_m_ = 1.21). Pooled fractions were diluted with 5 vol PBS, and MP_30_ pelleted by ultracentrifugation for 2 h at 30,000×*g* and 4°C, and MP_100_ pelleted by ultracentrifugation for 2 h at 100,000×*g* and 4°C. Pelleted MPs were resuspended in 200 µl PBS, further analyzed by flow cytometry, and stored at 4°C before use in our experiments of MP-mediated cell transfer of GFP-CFTR protein.

### MP Titration

#### (i) Titer in total MPs

Quantification of the total MP content in samples resuspended in PBS was determined by flow cytometry, using a FACSCanto™ II cytometer and the DIVA6 software (Becton Dickinson Biosciences). The CountBright™Absolute Counting Beads kit (Invitrogen Catalog # C-36950) and the Flow Cytometry Size Calibration kit (Nonfluorescent Microspheres; Invitrogen Catalog # F-13838) were used for the calibration of MP number and size, respectively. A total of 20,000 events was acquired for each sample for the calculation of the titer in total MPs.

#### (ii) Titer in bioactive MPs: MP-CAR, MP-CD46 and MP-GFP-CFTR

The proportion of MPs which displayed CAR, CD46 or GFP-CFTR at their surface and were potential conveyors of functional molecules was also determined by flow cytometry, using CAR or CD46 antibodies. For MP-GFP-CFTR, both GFP signal of the tagged CFTR protein and anti-CFTR antibody were used for flow cytometry analysis. The titer in CAR-, CD46 or GFP-positive MPs/ml was the one taken into account for MP-mediated cell transduction.

### MP-mediated Transfer of CAR, CD46 or GFP-CFTR into Target Cells

Samples of CHO cell monolayers (5×10^5^ cells/well) were incubated with 200 µl-aliquots of MP-CAR, MP-CD46 or MP-GFP-CFTR suspensions in PBS mixed with prewarmed serum-free medium, at a constant transducing dose of 5 MPs per cell. After 2 h MP-cell interaction at 37°C, the 200 µl-mix was removed and replaced by 500 µl of prewarmed complete medium. The cells were further incubated for 48 h at 37°C, and assayed for newly acquired functions, i.e. permissiveness to adenovirus, resistance to complement induced apoptosis, GFP signal, or chloride channel activity. MP-transduced cells were taken at 72 h after MP interaction, and incubated with HAdV5-GFP or HAdV5F35-GFP at 500 vp/cell. GFP-expression was monitored in live cells using a Zeiss Axiovert-135 inverted microscope (magnification: ×20) equipped with an AxioCam digital camera. Cells were then harvested at 48 h pi, and GFP expression quantitated using flow cytometry.

### CD46 Anti-complement Activity

MPs isolated from the culture medium of CHO cells (negative control MP-CHO) or CHO-CD46 (MP-CD46) were incubated with aliquots of recipient CHO cells for 2 h at 37°C and 5% CO_2_, as above described. The culture medium was then removed and replaced by fresh medium containing complement fraction C3 (Sigma-Aldrich) at the concentrations of 1, 10, and 100 µg/mL, and cells further incubated for 48 h. The degree of cell apoptosis was measured by flow cytometry, using the Annexin V-FITC apoptosis detection kit (Sigma-Aldrich) according to the manufacturer’s instructions.

### PCR Analysis

#### (i) Real-time RT-PCR quantification of GFP-CFTR-encoding mRNA

Total RNA was extracted from MPs released from GFP-CFTR-expressing cells, or from MP-GFP-CFTR-transduced cells, using the Nucleospin RNA II kit (Macherey Nagel). Aliquots of 1 µg RNA was reverse transcribed using the SuperScript™ III First Strand Synthesis SuperMix kit (Invitrogen) and real-time PCR was performed using the LightCycler and a LightCycler DNA Master SYBR Green I kit (Roche). Quantitative real-time PCR was performed using an antisense primer designed from the 5′ end of the CFTR gene (nucleotide position 78 in the CFTR gene; 5′-GCGCTGTCTGTATCCTTTCCTCAA) and a forward primer designed from the 3′ end of the *GFP* gene (nucleotide position 621; 5′-AACGAGAAGCGCGATCACATG). The PCR-amplified fragment was 196 nucleotides in length and overlapped the GFP and CFTR junction sequence [66].

#### (ii) GFP-CFTR DNA

The *GFP-CFTR* gene carried by the pCEP_4_-GFP-CFTR plasmid vector was detected using the same primers as above, on the host cell genomic DNA substrate obtained using the DNeasy Blood and Tissue kit (Qiagen).

### CFTR Channel Function Assayed by Membrane Potential-sensitive Oxonol Probe and Cell Imaging

The fluorescent voltage sensitive probe bis(1,3-dialkylthiobarbituric acid)oligomethine oxonol (DiSBAC_2_(3)) was used as previously described [65]. In brief, cells were loaded for 30 min with 100 nM DiSBAC2(3) in a normal chloride solution containing 136 mM NaCl, 4 mM KCl, 1 mM CaCl_2_, 1 mM MgCl_2_, 2.5 mM glucose and 10 mM Hepes (pH 7.4). Cells were then superfused with DiSBAC_2_(3) in a low chloride solution, whereby NaCl was replaced by sodium gluconate, and supplemented with a cAMP cocktail consisted of 200 µM dibutyryl-adenosine 3′:5′-cyclic monophosphate, 200 µM of 4-chlorophenylthio)-cAMP, 20 µM Forskolin and 50 µM 3-isobutyl-1-methylxanthine. Fluorescent cells were viewed on an inverted TMD300 microscope (Nikon AG, Kürsnacht, Switzerland) equipped with a high-sensitivity black and white CoolSNAP HQ2 CCD camera (Visitron Systems GmbH, Puchheim, Germany). DiSBAC2(3) was excited at 546 nm with a 100-W xenon lamp and the emitted fluorescence was collected through a 580 nm barrier filter. Images were captured every 10 sec, stored and processed using Metafluor version 8.01 software (Universal Imaging Corp., Downington, PA). Regions of interest were delineated for up to 30 cells and changes in the fluorescent signal measured in each region were expressed as the F_t_/F_0_ ratio, in which F_t_ and F_0_ were the fluorescence values at the time *t* and at the time when the cAMP cocktail was added, respectively. The cell-permeable glycinyl hydrazone compound (GlyH-101; Calbiochem) was used as a selective inhibitor of CFTR at 20 µM.

### Chemicals and Antibodies

Cycloheximide was purchased from Boehringer (Mannheim, Germany). DiSBAC_2_(3) was purchased from Invitrogen. Monoclonal antibodies against VSV-G (clone P5D4), human CD46 (clone 122-2) and human CAR (clone 3C100) were purchased from Santa Cruz Biotechnology. Polyclonal antiserum against human, mouse, rat, bovine, equine, canine and porcine TSG101 (ref. M-19, from goat), and against human, mouse, rat, canine and porcine CD63 (ref. H-193, from rabbit) were also purchased from Santa Cruz Biotechnology. Alexa Fluor® 568-conjugated goat anti-mouse IgG antibody were purchased from Invitrogen. The 10-nm colloidal gold-tagged goat anti-mouse IgG antibody was purchased from British Biocell International Ltd (Cardiff, UK). The anti-human CFTR antibody (LS-C14758-LSBio; LifeSpan-Biosciences, UK) was a mouse monoclonal IgM directed against an epitope located within the first extracellular loop, spanning amino acid residues 103–117. Alexa Fluor® 568-conjugated goat anti-mouse IgM was purchased from Invitrogen.

### Viral Vectors

#### (i) Adenoviral vectors

HAdV5-GFP and HAdV5F35-GFP vectors have been described in previous studies [36], [55], [64]. The capsid of HAdV5F35-GFP consisted of hexon and penton base capsomers of HAdV5, and of chimeric fibers made up of the shaft and knob domains of serotype 35 fiber (F35) fused to HAdV5 fiber tail. Chimeric vector HAdV5F35-GFP-CFTR has been previously described [66]. HAdV5F35-GFP-CFTR encoded the wild-type (wt) allele of the *CFTR* gene fused to the 3′ end of the *GFP* gene. Vector stocks were produced and titrated on HEK-293 cell monolayers [82].

#### (ii) Baculoviral vector AcMNPV-VSV-G

The coding sequence for the glycoprotein G of vesicular stomatitis virus (VSV-G) was inserted into the genome of *Autographa californica* MultiCapsid NucleoPolyhedrosis Virus (AcMNPV) in the polyhedrosis gene locus, under the control of the CMV immediate-early promoter. The infectious titer was determined by the plaque assay method in Sf9 cells, and expressed as plaque-forming units per mL (pfu/mL). The titer in physical virus particles of AcMPV-VSV-G vector was determined by adsorbance measurement at 260 nm (*A* ^260^) on 1-mL samples of SDS-denatured virions (0.1% SDS for 1 min at 56°C) in 1-cm pathlength cuvette, using the following formula : *A* ^260^ of 1.0 = 0.3×10^12 ^vp/mL, considering the length of 134 kbp for the viral genomic DNA. Infectious titers of stocks of AcMPV-VSV-G concentrated by ultracentrifugation were usually 5×10^9^ to 1×10^10^ pfu/mL, and the corresponding physical particle titers ranged between 1×10^12^ and 5×10^12 ^vp/mL [36].

### Electron Microscopy

#### (i) Negative staining of MPs

Samples were applied to carbon-coated grid and negatively stained with 1% uranyl acetate, pH 7.5. They were examined under a Jeol JEM-1400 electron microscope (EM), equiped with an ORIUS™ digital camera (Gatan France, 78113-Grandchamp).

#### (ii) Immunogold staining of MPs

Samples of MP suspension (10 µl) were deposited on top of carbon-coated grids. 30 sec later, the excess of liquid was removed by blotting with filter paper. 10 µl of a 50-fold diluted solution of primary antibody (anti-CAR or anti-CD46) was placed on the grid and incubated for 2 min at room temperature. The antibody solution was then removed by filter paper adsorption, and replaced by 10 µl Tris-buffered saline (TBS). After three steps of rinsing with TBS, grids were post-incubated with 10-nm colloidal gold-conjugated goat anti-mouse IgG antibody (British BioCell International, Cardiff, UK; diluted to 1:50 in TBS) for 2 min at room temperature. The secondary antibody solution was then removed by filter paper adsorption, and replaced by 10 µl of stain (2% uranyl acetate, pH 7.4). After a further 1 min, the grid was dried on filter paper, and examined under the EM as above.

#### (iii) EM analysis of MP-adenovirus complexes and cell sections

Samples of MPs incubated with adenoviral vectors, with or without postincubation with target CHO cells, were fixed with 2% glutaraldehyde in 0.1 M sodium cacodylate buffer (pH 7.4), pelleted, and post-fixed with osmium tetroxide (1% in 0.1 M cacodylate buffer, pH 7.4). The specimens were dehydrated and embedded in Epon resin and sectioned. Sections were stained with 7% uranyl acetate in methanol, post-stained with 2.6% alkaline lead citrate in H2O, and examined under the EM as above.

### Statistics

Results were expressed as mean ± SEM of *n* observations. Sets of data were compared with an analysis of variance (ANOVA) or a Student’s *t* test. Differences were considered statistically significant when *P*<0.05. Symbols used in figures were (*) for *P*<0.05, (**) for *P*<0.01, (***) for *P*<0.001, and ns for no significant difference, respectively. All statistical tests were performed using GraphPad Prism version 4.0 for Windows (Graphpad Software).

## Supporting Information

Figure S1 **Schematic procedure for MP isolation and fractionation.**
(TIFF)

Figure S2 **Influence of VSV-G-pseudotyping on the efficiency on the efficiency of MP-mediated transfer of CAR and CD46. (a),** The effect of the fusiogenic VSV-G glycoprotein on the efficiency of CAR transfer was evaluated by the degree of CHO permissiveness to the HAdV5-GFP vector. Aliquots of CHO cells were incubated with MP_30_CAR at different MP doses per cell, as indicated in the *x*-axis. At 72 h after MP_30_CAR interaction, cells were infected with HAdV5-GFP vector. Adenoviral vector HAdV5F35-GFP, which does not recognize CAR as cellular receptor, was used as the negative control. **(b),** The effect of VSV-G on the efficiency of CD46 transfer was evaluated by the degree of CHO permissiveness to the HAdV5F35-GFP vector. Aliquots of CHO cells were incubated with MP_30_CD46 at different MP doses per cell, as indicated in the *x*-axis. At 72 h after MP_30_CD46 interaction, cells were infected with HAdV5F35-GFP vector. Adenoviral vector HAdV5-GFP, which does not recognize CD46 as cellular receptor, was used as the negative control. In (a) and (b), MP_30_ lacking CAR or CD46 and carrying VSV-G alone were used as negative controls for CAR and CD46 receptor activity, respectively.
(EPS)

Figure S3 **Cellular localization of GFP-tagged CFTR glycoprotein in MP-donor and MP-recipient CHO cells. (A), MP-donor cells.** CHO cells expressing the GFP-CFTR fusion glycoprotein from the pCEP_4_-GFP-CFTR episomal plasmid were examined in confocal fluorescence microscopy. **(B), MP-recipient CHO cells.** CHO cells harvested at day-5 after interaction with MP_30_-GFP-CFTR were examined as above. (**a**), Cell observed in the GFP channel. (**b**), Merging of image (a) and DAPI staining. Scale bar, 10 µm.
(TIFF)

Figure S4 **DNA analysis of MP-GFP-CFTR-recipient cells.** CHO cells were harvested at day-5 after interaction with the following MP subclasses: MP_30_-LD (lane 1), MP_30_-ID (lane 2), MP_30_-HD (lane 3), MP_100_-LD (lane 4), MP_100_-ID (lane 25), MP_100_-HD (lane 6). DNA was extracted and analysed by PCR for the possible occurrence of pCEP4-GFP-CFTR, visualized by a specific 196-nt fragment. DNA from CHO-CD46 infected with Ad5F35-GFP-CFTR was used as positive control (lane 7). Lane 8, 1kb-DNA ladder (Fermentas).
(TIFF)
