# Supplementary figures and images for: Microparticle-Mediated Transfer of the Viral Receptors CAR and CD46, and the CFTR Channel in a CHO Cell Model Confers New Functions to Target Cells
Source: PLoS One. 2012 Dec 20;7(12):e52326. doi: 10.1371/journal.pone.0052326 (PMC3527531; doi:10.1371/journal.pone.0052326)

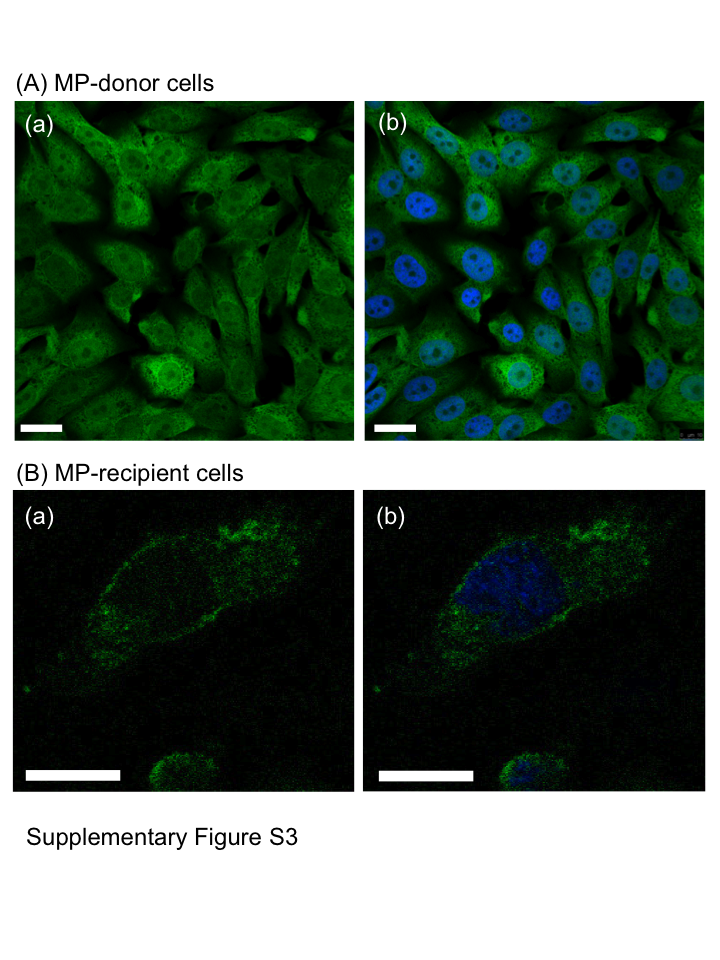

Supplement: Figure S3 — Cellular localization of GFP-tagged CFTR glycoprotein in MP-donor and MP-recipient CHO cells. (A), MP-donor cells. CHO cells expressing the GFP-CFTR fusion glycoprotein from the pCEP4-GFP-CFTR episomal plasmid were examined in confocal fluorescence microscopy. (B), MP-recipient CHO cells. CHO cells harvested at day-5 after interaction with MP30-GFP-CFTR were examined as above. (a), Cell observed in the GFP channel. (b), Merging of image (a) and DAPI staining. Scale bar, 10 µm. (TIFF) [file pone.0052326.s003.tiff]
